# Ureterosciatic hernia with concomitant Amyand hernia: Case report and review of the literature

**DOI:** 10.1016/j.radcr.2021.07.050

**Published:** 2021-08-17

**Authors:** Emma Sechrist, Alaa Elmaoued, Chiew-Jen Ong, Surbhi Trivedi, Marielia Gerena, Robert Wagner, Emad Allam

**Affiliations:** a Department of Radiology, Loyola University Medical Center, 2160 S 1st Ave, Maywood, IL 60153, USA; b Department of Radiology, University of Illinois at Chicago, 1740 W Taylor St, Chicago, IL 60612, USA

**Keywords:** Ureterosciatic hernia, Sciatic hernia, Ureteral hernia, Lindbom hernia, Amyand hernia, Inguinal hernia, BMI, body mass index, COPD, chronic obstructive pulmonary disease, GERD, gastroesophageal reflux disease, FDG, fluorodeoxyglucose

## Abstract

Ureterosciatic hernias are extremely rare, with fewer than 40 cases reported in the literature. We present a case of a patient with concurrent right ureterosciatic hernia (Lindbom hernia), ipsilateral bladder hernia, and appendix-containing inguinal hernia (Amyand hernia). These findings were discovered incidentally on imaging and the patient had no associated symptoms.

## Background

Ureterosciatic hernias, also referred to as Lindbom hernias, are extremely rare with fewer than 40 published cases [1], [2]. However, ureterosciatic hernias were the second most common type of sciatic hernia following ovary-containing sciatic hernias out of 97 reported cases from 1900 to 2008 [3]. Other typical sciatic hernia sac contents include the small intestine, colon, and urinary bladder. Ureterosciatic hernia occurs when the ureter and the retroperitoneum protrude through either the greater or lesser sciatic foramen. The “curlicue ureter” sign is considered pathognomonic for ureterosciatic hernia (Fig. 1) [4].

**Fig. 1(a):**
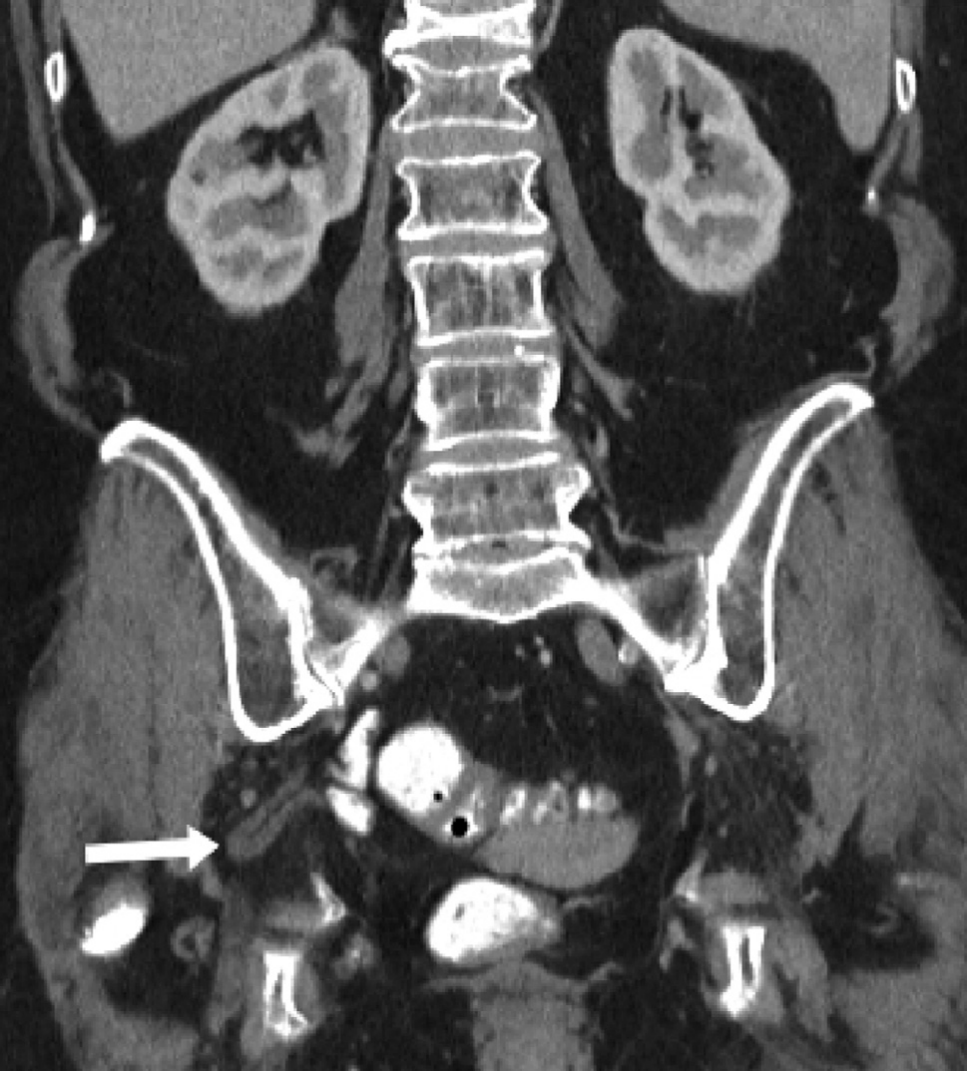
Coronal CT image in the nephrographic phase demonstrates a right ureterosciatic hernia with looping consistent with the curlicue sign (white arrow). Chronic compression fractures of the T12 and L1 vertebrae are also present.

Sciatic hernias are divided into three subtypes based on their location. The greater sciatic foramen is generally subdivided by the piriformis muscle into suprapiriform and infrapiriform spaces. Therefore, hernias through the greater sciatic foramen are termed suprapiriform or infrapiriform sciatic hernias. Hernias through the lesser sciatic foramen emerge between the sacrospinous and sacrotuberous ligaments, and are therefore classified as spinotuberous hernias [3].

The pathogenesis of sciatic hernias is not well understood. Unlike other types of hernias, the role of collagen metabolism has not been elucidated, likely due to its rarity [3]. A sciatic hernia may be the sequela of piriformis muscle atrophy, increased intra-abdominal pressure related to pregnancy, severe constipation, surgery, trauma, neuromuscular weakness, or hip pathology [5], [6]. It is often acquired but can be congenital. Females, particularly elderly females, are reportedly at greater risk which may be due to a wider pelvis or prior pregnancy [6]. Management and prognosis of this condition are uncertain due to its rarity and depend on the presenting symptomatology.

## Case presentation

**Fig. 2:**
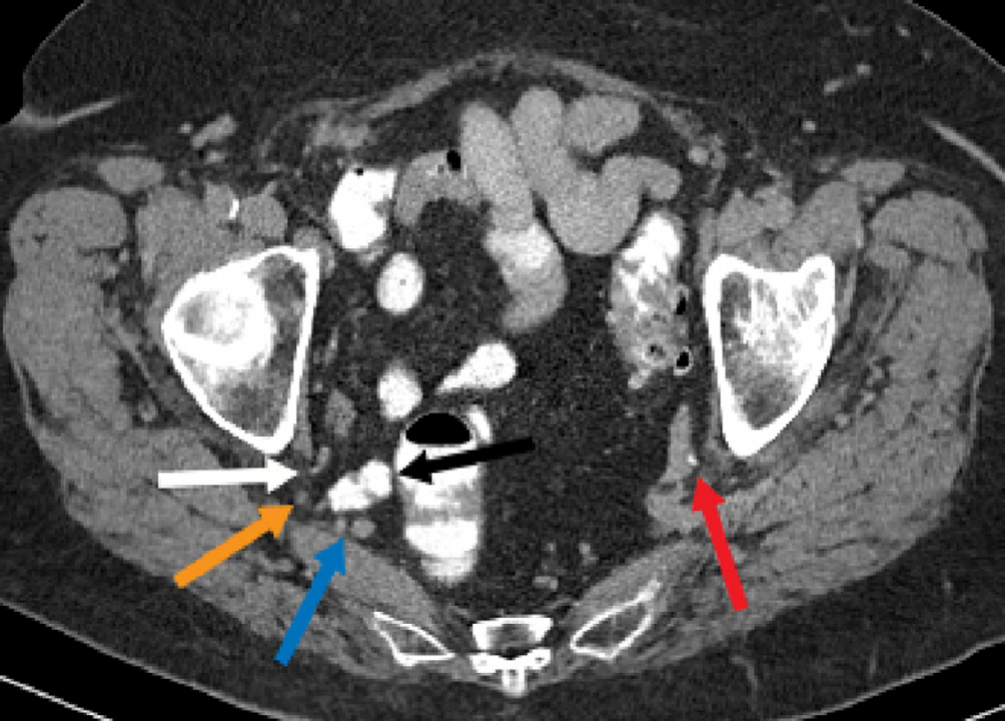
Axial CT image in the delayed phase shows the right ureter (white arrow) curving into the greater sciatic foramen in the infrapiriformis region. The sciatic nerve (orange arrow) and inferior gluteal vessels (blue arrow) are also seen at this infra-piriformis level. There is slight protrusion of the small bowel (black arrow) into the greater sciatic foramen. The left ureter (red arrow) has a somewhat posterior course near the sciatic notch, but subsequently coursed anteriorly towards the bladder (color version of figure is available online).

**Fig. 3:**
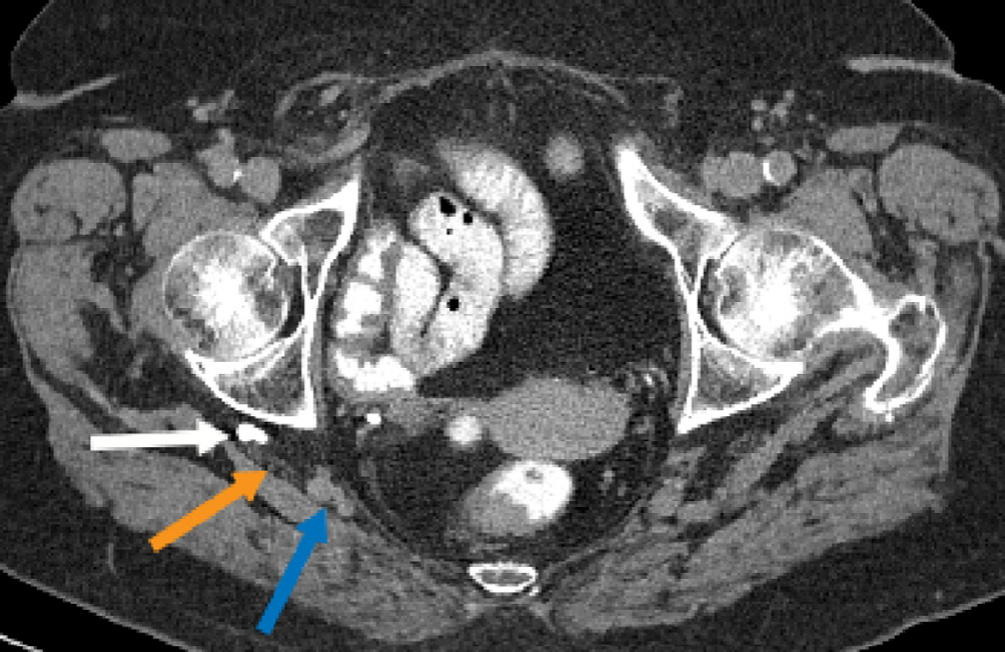
Axial CT image in the delayed phase shows a tubular structure opacified with contrast posterior to the right acetabulum, consistent with a right ureterosciatic hernia (white arrow). This abuts the right sciatic nerve (orange arrow). The inferior gluteal vessels are medial to this (blue arrow) (color version of figure is available online).

**Fig. 4:**
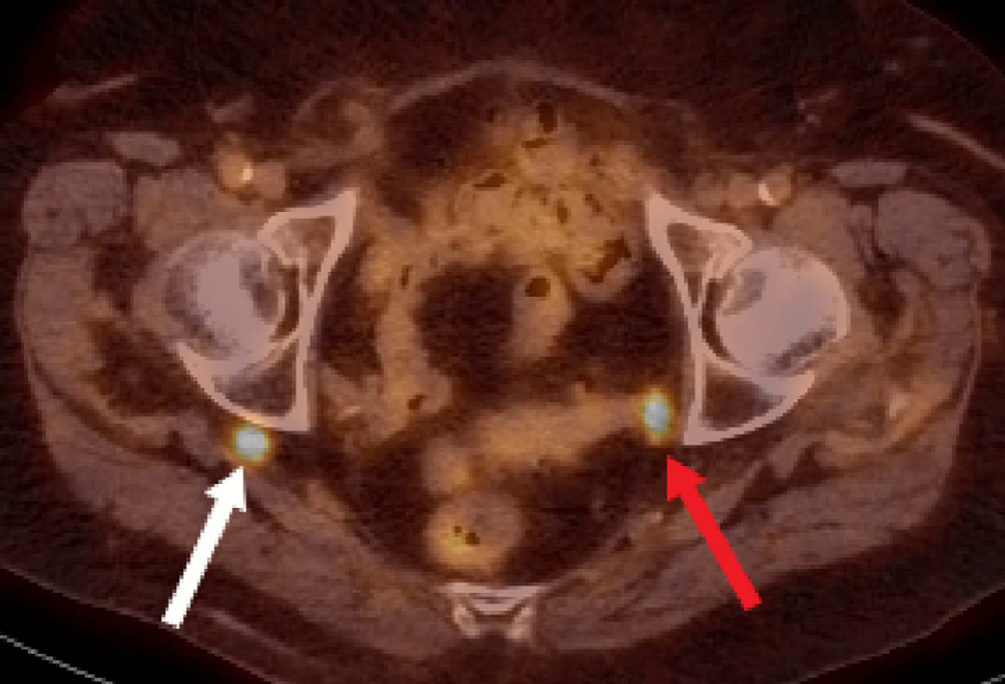
Axial PET/CT image demonstrates physiologic FDG uptake posterior to the right acetabulum (white arrow). When correlated with CT, this corresponds to the herniated right ureter. There is also physiologic FDG uptake in the left ureter (red arrow) (color version of figure is available online).

A 75-year-old woman with a history of obesity (BMI of 30), hyperlipidemia, osteoporosis, GERD, smoking and COPD presented with shortness of breath. CT revealed an incidental pulmonary nodule. Subsequent PET/CT examination for evaluation of the pulmonary nodule demonstrated increased FDG uptake posterior to the right acetabulum (Fig. 4). On review of prior CT examinations, including a CT urogram, this focus of FDG uptake corresponded to the right ureter which herniated through the greater sciatic foramen in the infrapiriformis region (Figs. 1, 2, and 3). There was slight protrusion of the small bowel into this hernia sac. Contralaterally, the left ureter had a posterior course but did not extend into the sciatic foramen (Fig. 2). There was mild, symmetric atrophy of the bilateral piriformis muscles.

**Fig. 5:**
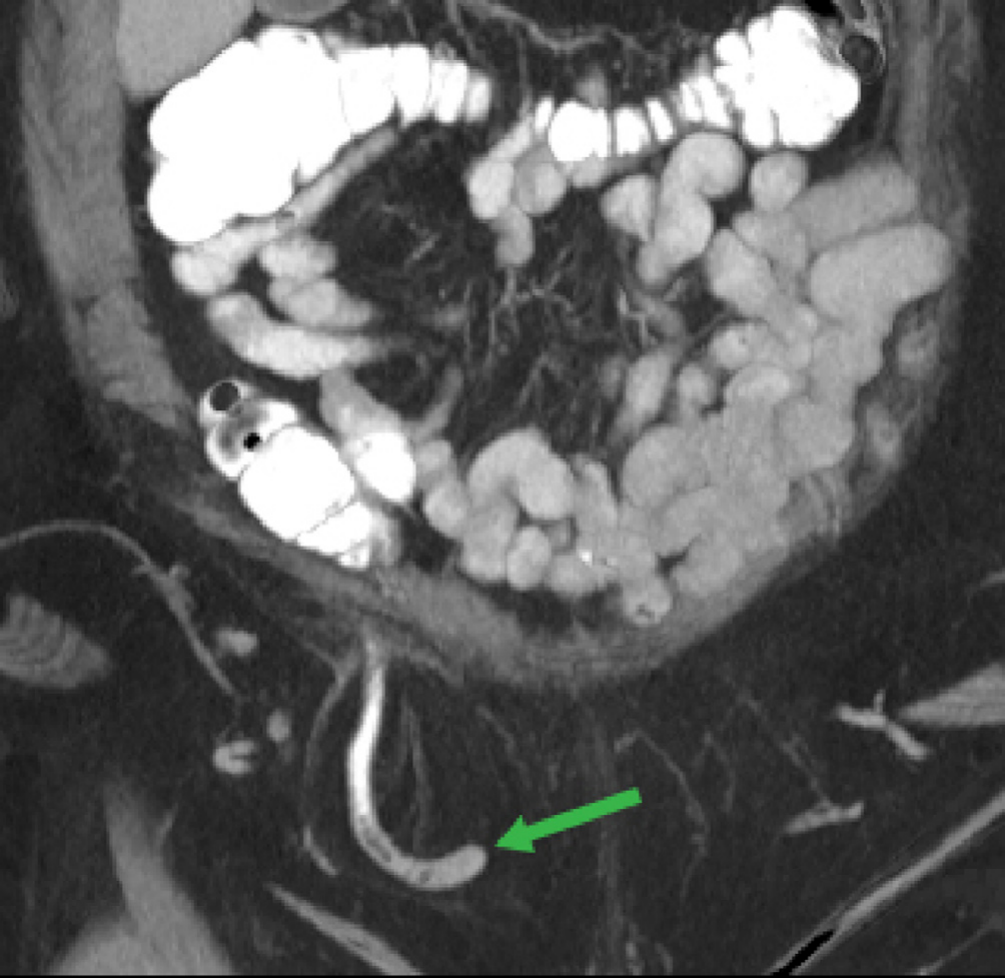
Coronal CT maximum intensity projection demonstrates a right inguinal hernia containing the appendix, consistent with an Amyand hernia (green arrow). Oral contrast is present in the bowel and appendix (color version of figure is available online).

**Fig. 6:**
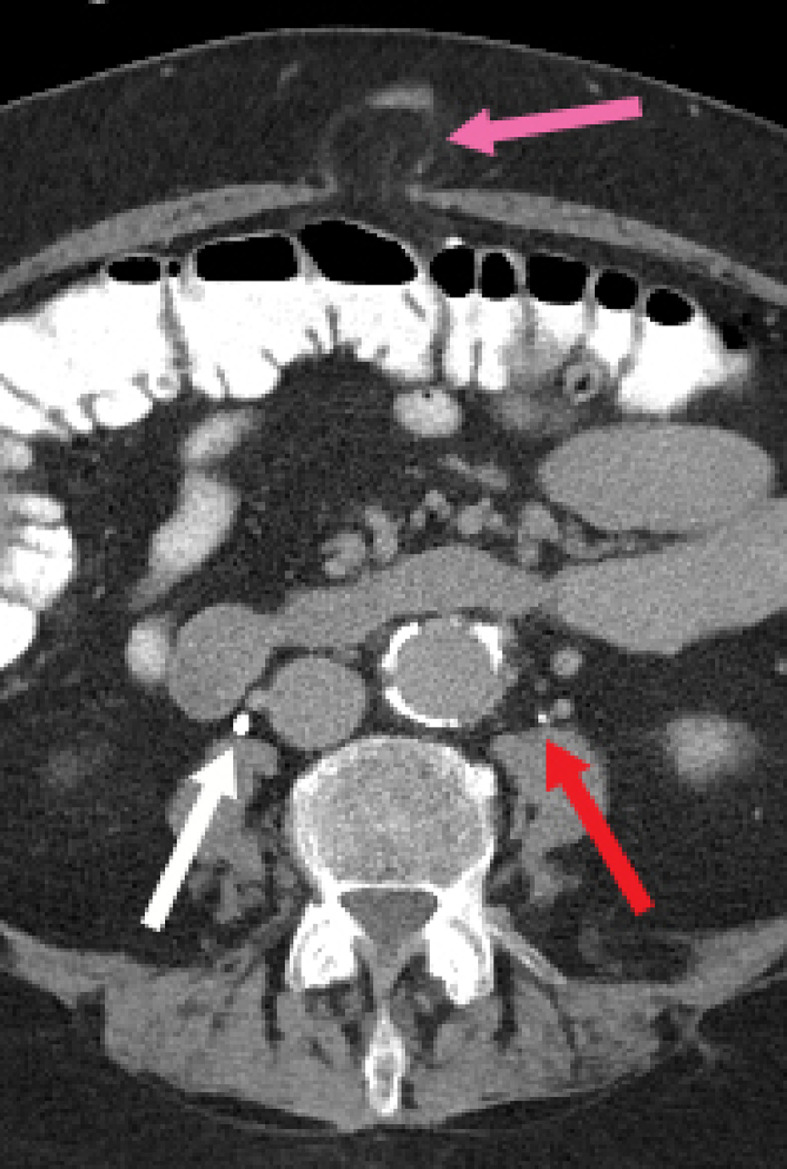
Axial CT image demonstrates a fat-containing umbilical hernia (pink arrow). The opacified ureters overlie the psoas muscles just inferior to the level of the kidneys (white and red arrows) (color version of figure is available online).

**Fig. 7:**
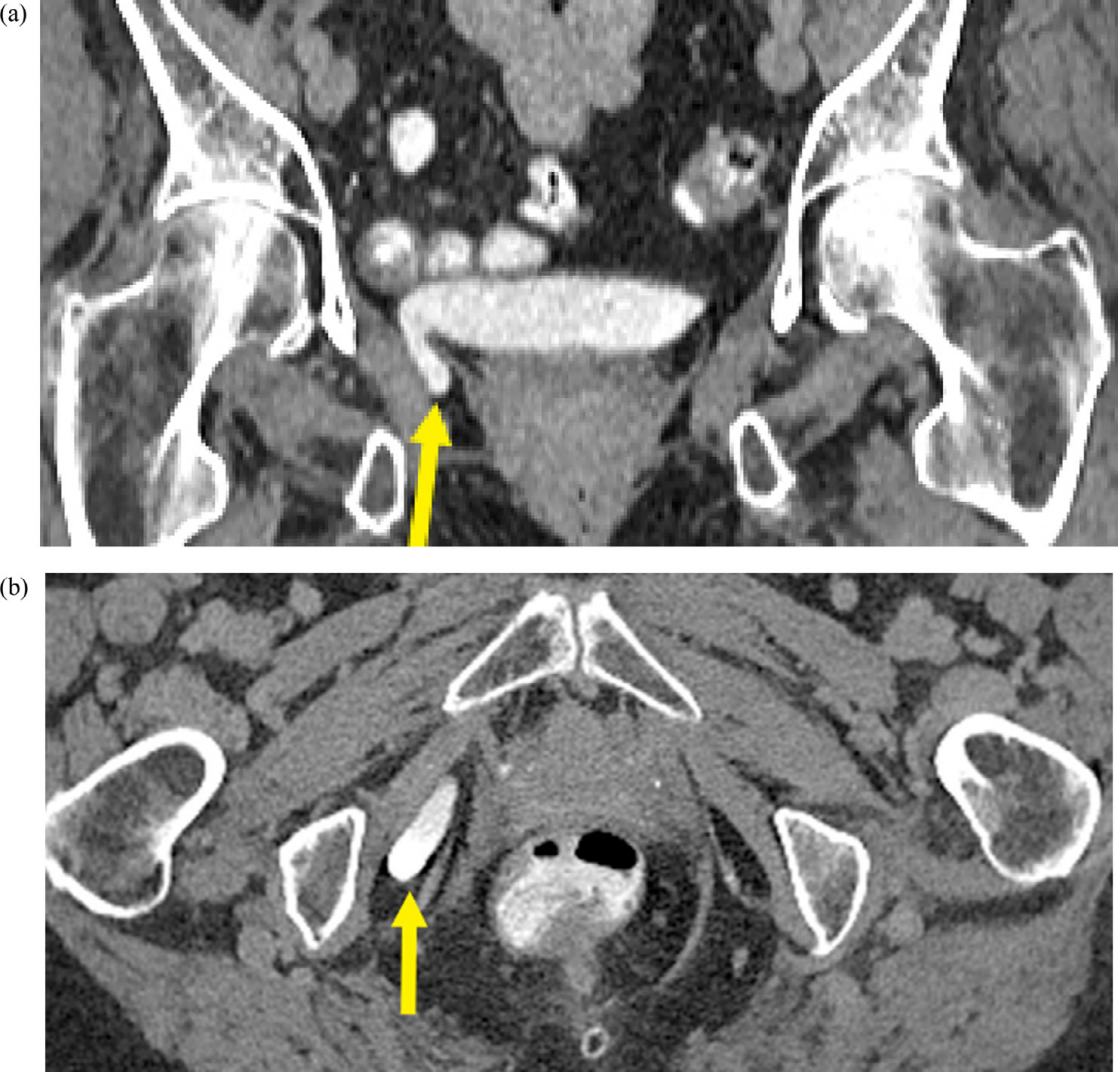
(a) Coronal and (b) axial CT images in the delayed phase demonstrate a right-sided bladder hernia containing contrast material (yellow arrow) extending through the pudendal canal (color version of figure is available online).

In addition to these findings, the CT demonstrated other concurrent hernias, including an Amyand hernia (Fig. 5) and a fat-containing umbilical hernia (Fig. 6). Nearly the entire appendix was seen within a right inguinal hernia, consistent with an Amyand hernia. Additionally, a right-sided bladder hernia was present which extended through the pudendal canal (Fig. 7). There were no signs of obstructive uropathy. The patient had no associated symptoms at the time of the PET/CT and had no history of abdominal or pelvic surgery.

## Discussion

Our case report depicts several concurrent hernias, including an extremely rare ureterosciatic hernia. This is the only published case of concurrent Lindbom and Amyand hernias. Pudendal or perineal herniation of the urinary bladder, as seen in this case, is also extremely rare [7]. It is unclear if there is an association between the development of a ureterosciatic hernia and other abdominopelvic hernias or collagen abnormalities. Of the limited reported cases of sciatic hernias, almost half of them were associated with significant comorbidities, including a few patients with coexisting hernias [3]. These hernias were often discovered incidentally on imaging. As illustrated in this case, ureteral hernias may be mistaken for neoplasm on PET/CT examination due to FDG uptake in an unusual location, and CT urography is helpful in clarifying the course of the ureter. MRI can be useful in delineating the relationship of the ureter with the sciatic nerve. Even with advanced imaging techniques, ureterosciatic hernias may evade detection because of intermittent reduction. In such cases, fluoroscopy may be more sensitive [3]. Due to its surgical implications, it is important to correctly report the location of the hernia, specifically whether it arises in the greater or lesser sciatic foramen, and its relationship to the adjacent neurovascular structures [8].

**Fig. 1(b):**
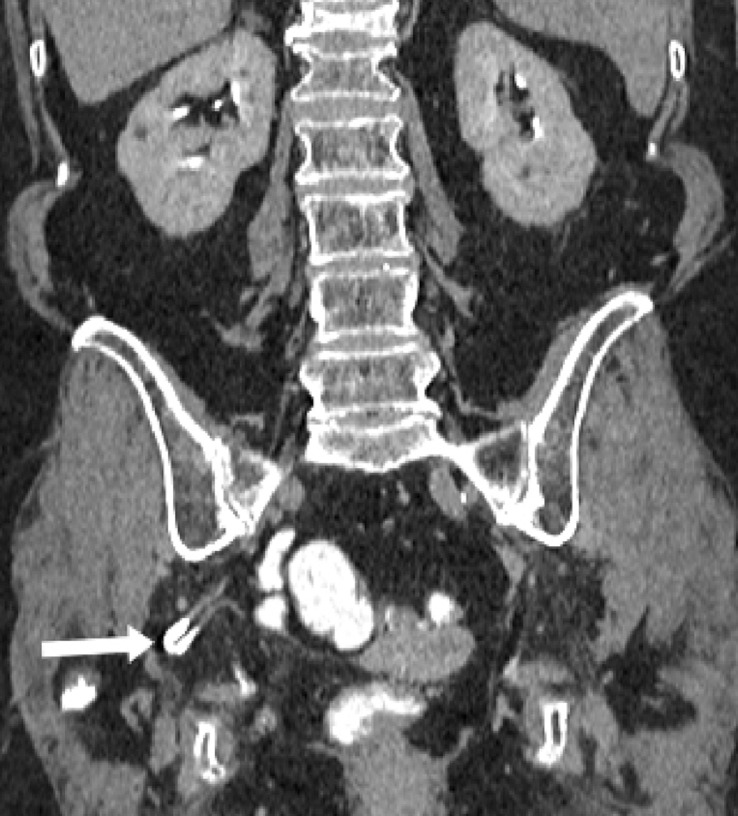
Coronal CT image in the delayed phase demonstrates a right ureterosciatic hernia with the curlicue sign (white arrow). The ureter is opacified with contrast in this image.

**Fig. 1(c):**
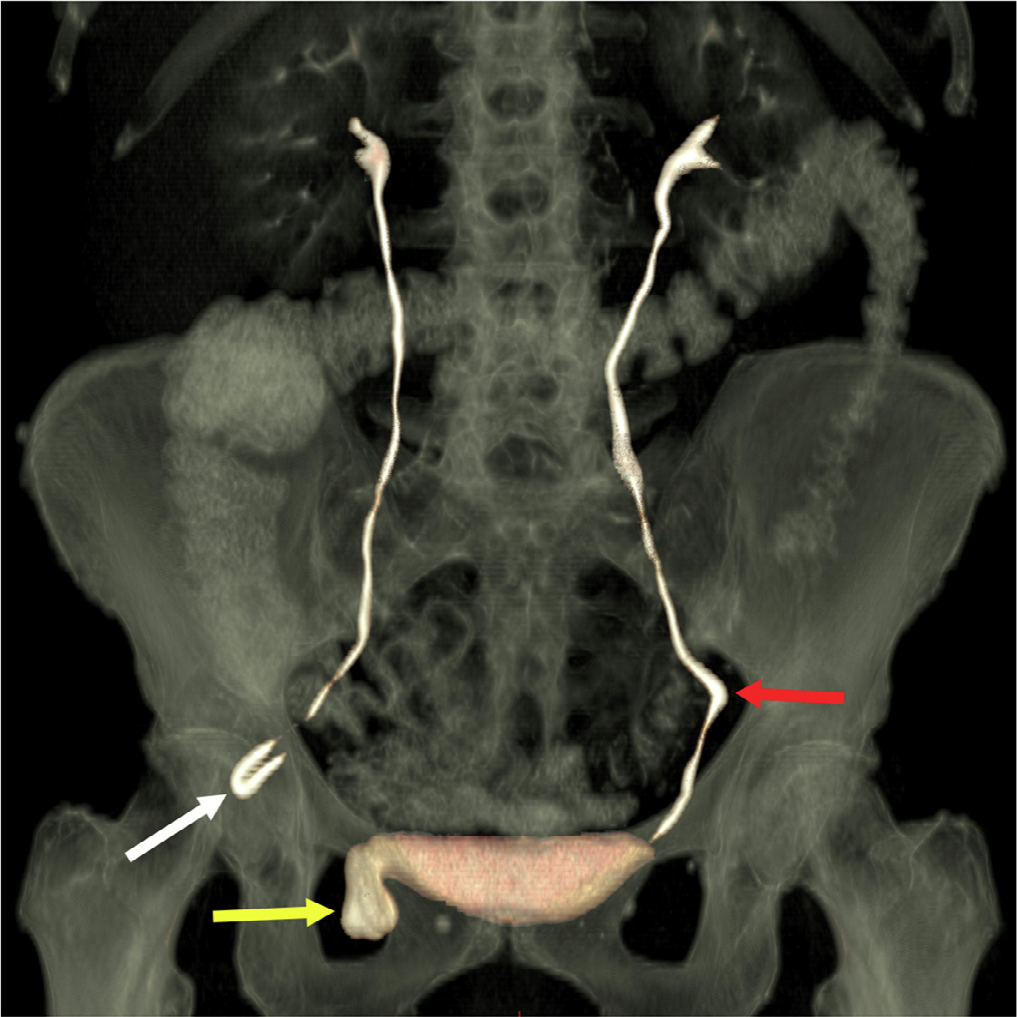
Coronal CT 3-D reconstruction with volume rendering demonstrating a right ureterosciatic hernia (white arrow), right bladder hernia (yellow arrow), and left ureteral deviation without herniation (red arrow) (color version of figure is available online).

Prior cases of asymptomatic ureterosciatic hernia have been followed with careful surveillance [9]. An aberrant course of the ureter is important to note, even if asymptomatic, as it may complicate other pelvic surgeries. For symptomatic cases including obstructive uropathy, surgical intervention is often warranted while considering patient comorbidities [2]. Surgical treatment may require excision of the hernia as well as reduction and reimplantation of the ureter and herniated sac. Other surgical methods include ureterotomy for antegrade or retrograde insertion of a ureteral stent, ureteropexy for ureter fixation, and sciatic hernioplasty to remove hernia defects [6]. Several complications are possible with the operative repair of ureterosciatic hernias, including ureteral and regional nerve injuries [6]. In a unique case from Japan in which the patient presented with flank pain and mild hydroureteronephrosis, manual transvaginal reduction of the sciatic hernia was successfully performed with ultrasound guidance from over the buttock [10].

## Conclusion

Ureterosciatic hernia is challenging to diagnose due to its rarity and nonspecific symptomatology. Awareness of this potentially hazardous anomaly and close attention for an aberrant course of the ureter on imaging studies is recommended [11]. Since the hernia is located in a complex anatomic space, immediate and delayed complications are possible with attempted surgical correction. Therefore, clinical and imaging surveillance of asymptomatic patients is advised rather than surgical correction. Precise guidelines for follow-up imaging have not been established.

## Patient consent

Patient consent: Formal consents are not required for the use of entirely anonymized images from which the individual cannot be identified - for example, x-rays, ultrasound images, pathology slides or laparoscopic images, provided that these do not contain any identifying marks and are not accompanied by text that might identify the individual concerned.
